# A general double-proximal gradient algorithm for d.c. programming

**DOI:** 10.1007/s10107-018-1292-2

**Published:** 2018-05-23

**Authors:** Sebastian Banert, Radu Ioan Boț

**Affiliations:** 1 Department of Mathematics, KTH Royal Institute of Technology, Lindstedtsvägen 25, 100 44 Stockholm, Sweden; 2 Faculty of Mathematics, University of Vienna, Oskar-Morgenstern-Platz 1, 1090 Vienna, Austria

**Keywords:** d.c. programming, Toland dual, Proximal-gradient algorithm, Kurdyka–Łojasiewicz property, Convergence analysis, 90C26, 49M29, 65K05

## Abstract

The possibilities of exploiting the special structure of d.c. programs, which consist of optimising the difference of convex functions, are currently more or less limited to variants of the DCA proposed by Pham Dinh Tao and Le Thi Hoai An in 1997. These assume that either the convex or the concave part, or both, are evaluated by one of their subgradients. In this paper we propose an algorithm which allows the evaluation of both the concave and the convex part by their proximal points. Additionally, we allow a smooth part, which is evaluated via its gradient. In the spirit of primal-dual splitting algorithms, the concave part might be the composition of a concave function with a linear operator, which are, however, evaluated separately. For this algorithm we show that every cluster point is a solution of the optimisation problem. Furthermore, we show the connection to the Toland dual problem and prove a descent property for the objective function values of a primal-dual formulation of the problem. Convergence of the iterates is shown if this objective function satisfies the Kurdyka–Łojasiewicz property. In the last part, we apply the algorithm to an image processing model.

## Introduction

Optimisation problems where the objective function can be written as a difference of two convex functions arise naturally in several applications, such as image processing [1], machine learning [2], optimal transport [3] and sparse signal recovering [4]. Generally, the class of d.c. functions is rather broad and contains for example every twice continuously differentiable function. For an overview over d.c. functions, see e.g. [5].

The classical approach to iteratively find local extrema of d.c. problems was described by Tao and An [6] in 1997 under the name DCA (d.c. algorithms). One of the most recent papers on this topic is [7], where an accelerated variant of the DCA method is proposed under the supplementary assumption that both the convex and the concave part are continuously differentiable. In 2003, Sun, Sampaio and Candido introduced a proximal point approach into the theory of d.c. algorithm [8], where the convex part is evaluated by its proximal point operator, while its concave part is still evaluated by one of its subgradients. Later on, the approach in [8] has been extended in [9–11] by considering in the convex part a further convex smooth summand that is evaluated via its gradient.

In this paper, we go one step further by proposing an algorithm, where both the convex and concave parts are evaluated via proximal steps. In convex optimisation, using proximal steps instead of subgradient steps has several advantages:

- The subdifferential at a point may be a non-singleton set, in particular it may be empty or may consist of several distinct elements. In an algorithm, one may get stuck or have to choose one, respectively.
- Even if the subdifferential is a singleton in each step, it might be highly discontinuous, so small deviations might lead to a very different behaviour of the iterations.
- Better convergence rates can be guaranteed for proximal algorithms than for subgradient algorithms (compare [12] and [13, Theorem 3.2.3]).

In addition, we consider a linear operator in the concave part of the objective function, which is evaluated in a forward manner in the spirit of primal-dual splitting methods.

In Sect. 2, we present the problem to be solved together with its Toland dual and attach to them a primal-dual formulation in the form of a minimisation problem, too. We derive first-order optimality conditions, relate the optimal solutions and the critical points of the primal-dual minimisation problems to the optimal solutions and, respectively, the critical points of both primal and dual optimisation problems.

In Sect. 3, we propose a double-proximal d.c. algorithm, which generates both a primal and a dual sequence of iterates and show several properties which make it comparable to DCA. More precisely, we prove a descent property for the objective function values of a primal-dual formulation and that every cluster point of the sequence of primal iterates is a critical point of the primal problem, while every critical point of the sequence of dual iterates is a critical point of the dual problem.

In Sect. 4, we show global convergence of our algorithm and convergence rates for the iterates in certain cases, provided that the objective function of the primal-dual reformulation satisfies the Kurdyka–Łojasiewicz property; in other words, it is a *KŁ function*. The convergence analysis relies on methods and concepts of real algebraic geometry introduced by Łojasiewicz [14] and Kurdyka [15] and later developed in the nonsmooth setting by Attouch, Bolte, Redont, and Soubeyran [16], Attouch, Bolte, and Svaiter [17] and Bolte, Sabach and Teboulle [18]. One of the remarkable properties of the KŁ functions is their ubiquity in applications (see [18]). The class of KŁ functions contains semi-algebraic, real sub-analytic, semiconvex, uniformly convex and convex functions satisfying a growth condition.

We close our paper with some numerical examples addressing an image deblurring and denoising problem in the context of different d.c. regularisations.

### Notation and preliminaries

For the theory of convex analysis in finite-dimensional spaces, see the book [19]. We shall consider functions taking values in the *extended real line*$\overline{\mathbb {R}}\mathrel {\mathop :}=\mathbb {R}\cup \left\{ +\infty , -\infty \right\}$. We agree on the order $-\infty< a < +\infty$ for any real number *a* and the operations

$$\begin{aligned}&+\infty + a = a + \infty = +\infty - \infty = -\infty + \infty = +\infty + \infty = +\infty , \\&-\infty + a = a - \infty = -\infty - \infty = -\infty , \\&0 \cdot \mathord {\left( -\infty \right) } = 0, 0 \cdot \mathord {\left( +\infty \right) } = +\infty \end{aligned}$$

for arbitrary $a \in \mathbb {R}$ (see [20]). Let $\mathcal {H}$ be a real finite-dimensional Hilbert space. For a function $f: \mathcal {H}\rightarrow \overline{\mathbb {R}}$, we denote by

$$\begin{aligned} {{\mathrm{dom}}}~ f \mathrel {\mathop :}=\left\{ x\in \mathcal {H} \,\Big \vert \, f\mathord {\left( x\right) } < +\infty \right\} \end{aligned}$$

its *domain*. The function *f* is called *proper* if it does not take the value $-\infty$ and ${{\mathrm{dom}}}~ f \ne \emptyset$. It is called *convex* if

$$\begin{aligned} f\mathord {\left( \mathord {\left( 1 - \lambda \right) } x + \lambda y\right) } \le \mathord {\left( 1 - \lambda \right) } f\mathord {\left( x\right) } + \lambda f\mathord {\left( y\right) } \end{aligned}$$

for all $x, y \in \mathcal {H}$ and $0 \le \lambda \le 1$. The *conjugate function*$f^*: \mathcal {H}\rightarrow \overline{\mathbb {R}}$ of $f: \mathcal {H}\rightarrow \overline{\mathbb {R}}$ is defined by

$$\begin{aligned} f^*\mathord {\left( x^*\right) } = \sup \left\{ \left\langle x^*, x \right\rangle - f\mathord {\left( x\right) } \,\Big \vert \, x\in \mathcal {H} \right\} . \end{aligned}$$

If *f* is proper, convex, and lower semicontinuous, then $$f^{**} \mathrel {\mathop :}=\mathord {\left( f^*\right) }^* = f$$ by the Fenchel–Moreau theorem.

The *convex subdifferential*$\partial f\mathord {\left( x\right) }$ at $x\in \mathcal {H}$ of a function $f: \mathcal {H}\rightarrow \overline{\mathbb {R}}$ is empty if $x \notin {{\mathrm{dom}}}~ f$ and

$$\begin{aligned} \partial f\mathord {\left( x\right) } = \left\{ x^* \in \mathcal {H} \,\Big \vert \, \forall y\in \mathcal {H}: f\mathord {\left( y\right) } \ge f\mathord {\left( x\right) } + \left\langle x^*, y - x \right\rangle \right\} \end{aligned}$$

otherwise. Let $\gamma > 0$ and $f: \mathcal {H}\rightarrow \overline{\mathbb {R}}$ be proper, convex, and lower semicontinuous. The *proximal point*${{\mathrm{Prox}}}_{\gamma f}\mathord {\left( x\right) }$ of $\gamma f$ at $x\in \mathcal {H}$ is defined as

$$\begin{aligned} {{\mathrm{Prox}}}_{\gamma f}\mathord {\left( x\right) } = {{\mathrm{arg\,min}}}\left\{ \gamma f\mathord {\left( y\right) } + \frac{1}{2} \left\| y - x \right\| ^2 \,\Big \vert \, y \in \mathcal {H} \right\} . \end{aligned}$$

The set of minimisers in the definition above is a singleton [21, Proposition 12.15], and the proximal point is characterised by the variational inequality [21, Proposition 12.26]

$$\begin{aligned} f\mathord {\left( y\right) } \ge f\mathord {\left( {{\mathrm{Prox}}}_{\gamma f}\mathord {\left( x\right) }\right) } + \frac{1}{2\gamma } \left\langle y - {{\mathrm{Prox}}}_{\gamma f}\mathord {\left( x\right) }, x - {{\mathrm{Prox}}}_{\gamma f}\mathord {\left( x\right) } \right\rangle \end{aligned}$$

for all $y\in \mathcal {H}$, which is equivalent to

1 $$\begin{aligned} \frac{1}{\gamma } \mathord {\left( x - {{\mathrm{Prox}}}_{\gamma f}\mathord {\left( x\right) }\right) } \in \partial f\mathord {\left( {{\mathrm{Prox}}}_{\gamma f}\mathord {\left( x\right) }\right) }. \end{aligned}$$

When dealing with nonconvex and nonsmooth functions, we have to consider subdifferentials more general than the convex one. The *Fréchet subdifferential*$\partial _F f\mathord {\left( x\right) }$ at $x\in \mathcal {H}$ of a proper and lower semicontinuous function $f: \mathcal {H}\rightarrow \overline{\mathbb {R}}$ is empty if $x \notin {{\mathrm{dom}}}~ f$ and

$$\begin{aligned} \partial _F f\mathord {\left( x\right) } = \left\{ x^* \in \mathcal {H} \,\Big \vert \, \liminf _{\begin{array}{c} y \rightarrow x \\ y \ne x \end{array}} \frac{f\mathord {\left( y\right) } - f\mathord {\left( x\right) } - \left\langle x^*, y - x \right\rangle }{\left\| y - x \right\| } \ge 0 \right\} \end{aligned}$$

otherwise. The *limiting (Mordukhovich) subdifferential*$\partial _L f\mathord {\left( x\right) }$ at $x\in \mathcal {H}$ of a proper and lower semicontinuous function $f: \mathcal {H}\rightarrow \overline{\mathbb {R}}$ is empty if $x\notin {{\mathrm{dom}}}~ f$, and

$$\begin{aligned} \partial _L f\mathord {\left( x\right) }= & {} \biggl \{x^* \in \mathcal {H}\, \bigg \vert \,\exists \mathord {\left( x_k\right) }_{k\ge 0}, \mathord {\left( x^*_k\right) }_{k\ge 0}: x_k \in \mathcal {H}, x^*_k \in \partial _F f\mathord {\left( x_k\right) }, k \ge 0, \\&x_k \rightarrow x, f\mathord {\left( x_k\right) } \rightarrow f\mathord {\left( x\right) }, x^*_k \rightarrow x^* \text { as } k\rightarrow +\infty \biggr \} \end{aligned}$$

otherwise.

## Problem statement

Let $\mathcal {G}$ and $\mathcal {H}$ be real finite-dimensional Hilbert spaces, let $g: \mathcal {H}\rightarrow \overline{\mathbb {R}}$ and $h: \mathcal {G}\rightarrow \overline{\mathbb {R}}$ be proper, convex, and lower semicontinuous functions, let $\varphi : \mathcal {H}\rightarrow \mathbb {R}$ be a convex, Fréchet differentiable function with $\frac{1}{\beta }$-Lipschitz continuous gradient, for some $\beta > 0$, and let $K: \mathcal {H}\rightarrow \mathcal {G}$ be a linear mapping (and $K^*: \mathcal {G}\rightarrow \mathcal {H}$ its adjoint). We consider the problem

2 $$\begin{aligned} \min \left\{ g\mathord {\left( x\right) } + \varphi \mathord {\left( x\right) } - h\mathord {\left( Kx\right) } \,\Big \vert \, x\in \mathcal {H} \right\} \end{aligned}$$

together with its Toland dual problem [22, 23]

3 $$\begin{aligned} \min \left\{ h^*\mathord {\left( y\right) } - \mathord {\left( g + \varphi \right) }^*\mathord {\left( K^* y\right) } \,\Big \vert \, y\in \mathcal {G} \right\} . \end{aligned}$$

The following primal-dual formulation will turn out to be useful in the sequel:

4 $$\begin{aligned} \min \left\{ \Phi \mathord {\left( x, y\right) } \,\Big \vert \, x \in \mathcal {H}, y\in \mathcal {G} \right\} \quad \text {with } \Phi \mathord {\left( x, y\right) } \mathrel {\mathop :}=g\mathord {\left( x\right) } + \varphi \mathord {\left( x\right) } + h^*\mathord {\left( y\right) } - \left\langle y, Kx \right\rangle , \end{aligned}$$

where $\Phi : \mathcal {H}\times \mathcal {G}\rightarrow \overline{\mathbb {R}}$ is proper and lower semicontinuous.

Let us derive necessary optimality conditions for the problems (2), (3), and (4):

### Proposition 1

1. The optimal values of (2), (3), and (4) are equal.
2. For all $x \in \mathcal {H}$ and $y \in \mathcal {G}$,
  $$\begin{aligned} \Phi \mathord {\left( x, y\right) }&\ge g\mathord {\left( x\right) } + \varphi \mathord {\left( x\right) } - h\mathord {\left( Kx\right) }\qquad \text {and} \\ \Phi \mathord {\left( x, y\right) }&\ge h^*\mathord {\left( y\right) } - \mathord {\left( g + \varphi \right) }^*\mathord {\left( y\right) } \mathord {\left( K^* y\right) }. \end{aligned}$$
3. Let ${\bar{x}} \in \mathcal {H}$ be a solution of (2). Then $\partial \mathord {\left( h\circ K\right) }\mathord {\left( {\bar{x}}\right) } \subseteq \partial g\mathord {\left( {\bar{x}}\right) } + \nabla \varphi \mathord {\left( {\bar{x}}\right) }$.
4. Let ${\bar{y}} \in \mathcal {G}$ be a solution of (3). Then $\partial \mathord {\left( \mathord {\left( g + \varphi \right) }^* \circ K^*\right) }\mathord {\left( {\bar{y}}\right) } \subseteq \partial h^*\mathord {\left( {\bar{y}}\right) }$.
5. Let $\mathord {\left( {\bar{x}}, {\bar{y}}\right) }\in \mathcal {H}\times \mathcal {G}$ be a solution of (4). Then ${\bar{x}}$ is a solution of (2), and ${\bar{y}}$ is a solution of (3). Furthermore, the inclusions
  5 $$\begin{aligned} K^* {\bar{y}}&\in \partial g\mathord {\left( {\bar{x}}\right) } + \nabla \varphi \mathord {\left( {\bar{x}}\right) }, \end{aligned}$$
  6 $$\begin{aligned} K{\bar{x}}&\in \partial h^*\mathord {\left( {\bar{y}}\right) } \end{aligned}$$
  hold.

### Proof

1. By the Fenchel–Moreau theorem, applied to *h*, we have
  $$\begin{aligned}&\inf \left\{ g\mathord {\left( x\right) } + \varphi \mathord {\left( x\right) } - h\mathord {\left( Kx\right) } \,\Big \vert \, x\in \mathcal {H} \right\} \\&\quad = \inf \left\{ g\mathord {\left( x\right) } + \varphi \mathord {\left( x\right) } - h^{**}\mathord {\left( Kx\right) } \,\Big \vert \, x\in \mathcal {H} \right\} \\&\quad = \inf \left\{ g\mathord {\left( x\right) } + \varphi \mathord {\left( x\right) } - \sup \left\{ \left\langle y, Kx \right\rangle - h^*\mathord {\left( y\right) } \,\Big \vert \, y\in \mathcal {G} \right\} \,\Big \vert \, x \in \mathcal {H} \right\} \\&\quad = \inf \left\{ g\mathord {\left( x\right) } + \varphi \mathord {\left( x\right) } + h^*\mathord {\left( y\right) } - \left\langle y, Kx \right\rangle \,\Big \vert \, x \in \mathcal {H}, y \in \mathcal {G} \right\} \\&\quad = \inf \left\{ h^*\mathord {\left( y\right) } - \sup \left\{ \left\langle x, K^* y \right\rangle - \mathord {\left( g + \varphi \right) }\mathord {\left( x\right) } \,\Big \vert \, x\in \mathcal {H} \right\} \,\Big \vert \, y \in \mathcal {G} \right\} \\&\quad = \inf \left\{ h^*\mathord {\left( y\right) } - \mathord {\left( g + \varphi \right) }^*\mathord {\left( K^* y\right) } \,\Big \vert \, y \in \mathcal {G} \right\} . \end{aligned}$$
2. Let $x \in \mathcal {H}$ and $y \in \mathcal {G}$. Then,
  $$\begin{aligned} g\mathord {\left( x\right) } + \varphi \mathord {\left( x\right) } - h\mathord {\left( Kx\right) }&= g\mathord {\left( x\right) } + \varphi \mathord {\left( x\right) } - h^{**}\mathord {\left( Kx\right) } \\&= g\mathord {\left( x\right) } + \varphi \mathord {\left( x\right) } - \sup \left\{ \left\langle Kx, {\tilde{y}} \right\rangle - h^*\mathord {\left( {\tilde{y}}\right) } \,\Big \vert \, {\tilde{y}} \in \mathcal {G} \right\} \\&\le g\mathord {\left( x\right) } + \varphi \mathord {\left( x\right) } - \left\langle Kx, y \right\rangle + h^*\mathord {\left( y\right) }, \end{aligned}$$
  and the other inequality is verified by an analogous calculation.
3. This kind of optimality condition is classical in d.c. programming, see e.g. [8, Proposition 1.1].
4. The proof of this statement is analogous.
5. Let $\mathord {\left( {\bar{x}}, {\bar{y}}\right) }$ be a solution of (4). (In particular, if such a solution exists, the common optimal value of (2), (3) and (4) must be finite.) The function $x \mapsto \Phi \mathord {\left( x, {\bar{y}}\right) }$ is convex and takes a minimum at ${\bar{x}}$. Therefore
  $$\begin{aligned} 0 \in \partial g\mathord {\left( {\bar{x}}\right) } + \nabla \varphi \mathord {\left( {\bar{x}}\right) } - K^* {\bar{y}}, \end{aligned}$$
  which proves (5). The same argument works for the function $y \mapsto \Phi \mathord {\left( {\bar{x}}, y\right) }$ and implies
  $$\begin{aligned} 0 \in \partial h^*\mathord {\left( {\bar{y}}\right) } - K{\bar{x}}, \end{aligned}$$
  which is (6). For these inclusions, we obtain equality in the Young–Fenchel inequality [21, Proposition 16.9], i.e.,
  $$\begin{aligned} \mathord {\left( g + \varphi \right) }^*\mathord {\left( K^* {\bar{y}}\right) } + \mathord {\left( g + \varphi \right) }\mathord {\left( {\bar{x}}\right) }&= \left\langle {\bar{x}}, K^* {\bar{y}} \right\rangle , \\ h^*\mathord {\left( {\bar{y}}\right) } + h\mathord {\left( K{\bar{x}}\right) }&= \left\langle {\bar{y}}, K{\bar{x}} \right\rangle . \end{aligned}$$
  Therefore, by subtracting these equalities,
  $$\begin{aligned} \mathord {\left( g + \varphi \right) }\mathord {\left( {\bar{x}}\right) } - h\mathord {\left( K{\bar{x}}\right) }&= h^*\mathord {\left( {\bar{y}}\right) } - \mathord {\left( g + \varphi \right) }^*\mathord {\left( K^* {\bar{y}}\right) } \\&= h^*\mathord {\left( {\bar{y}}\right) } - \sup \left\{ \left\langle x, K^*{\bar{y}} \right\rangle - g\mathord {\left( x\right) } - \varphi \mathord {\left( x\right) } \,\Big \vert \, x\in \mathcal {H} \right\} \\&\le h^*\mathord {\left( {\bar{y}}\right) } + g\mathord {\left( {\bar{x}}\right) } + \varphi \mathord {\left( {\bar{x}}\right) } - \left\langle {\bar{x}}, K^* {\bar{y}} \right\rangle . \end{aligned}$$
  Since $\mathord {\left( {\bar{x}}, {\bar{y}}\right) }$ is a solution of (4), the last expression equals the common optimal value of (2), (3), and (4). $\square$

### Definition 1

We say that $\mathord {\left( {\bar{x}}, {\bar{y}}\right) } \in \mathcal {H}\times \mathcal {G}$ is a *critical point* of the objective function $\Phi$ of (4) if the inclusions (5) and (6) are satisfied. We denote by $\mathrm {crit}\Phi$ the set of critical points of the function $\Phi$.

### Remark 1

If $\mathord {\left( {\bar{x}}, {\bar{y}}\right) } \in \mathcal {H}\times \mathcal {G}$ is a critical point of $\Phi$, then

7 $$\begin{aligned} K^* {\bar{y}}&\in K^* \partial h\mathord {\left( K{\bar{x}}\right) } \cap \mathord {\left( \partial g\mathord {\left( {\bar{x}}\right) } + \nabla \varphi \mathord {\left( {\bar{x}}\right) }\right) }, \end{aligned}$$

8 $$\begin{aligned} K {\bar{x}}&\in K \partial \mathord {\left( g + \varphi \right) }^* \mathord {\left( K^* {\bar{y}}\right) } \cap \partial h^*\mathord {\left( {\bar{y}}\right) }. \end{aligned}$$

By adopting the terminology of e.g. [6, p. 297], we denote by

$$\begin{aligned} \mathrm {crit}\mathord {\left( g + \varphi - h \circ K\right) }:= \{x \in \mathcal {H}: K^* \partial h\mathord {\left( K x\right) } \cap \mathord {\left( \partial g\mathord {\left( x\right) } + \nabla \varphi \mathord {\left( x\right) }\right) } \ne \emptyset \} \end{aligned}$$

the set of critical points of the objective function $g + \varphi - h \circ K$ of (2) and by

$$\begin{aligned} \mathrm {crit}\mathord {\left( h^* - \mathord {\left( g + \varphi \right) }^* \circ K^*\right) }:= \{y \in \mathcal {G}: K \partial \mathord {\left( g + \varphi \right) }^* \mathord {\left( K^*y\right) } \cap \partial h^*\mathord {\left( y\right) } \ne \emptyset \} \end{aligned}$$

the set of critical points of the objective function $h^* - \mathord {\left( g + \varphi \right) }^* \circ K^*$ of (3). (Recall that $K^* \partial h\mathord {\left( Kx\right) } \subseteq \partial \mathord {\left( h \circ K\right) }\mathord {\left( x\right) }$ and $K \partial \mathord {\left( g + \varphi \right) }^* \mathord {\left( K^*y\right) } \subseteq \partial \mathord {\left( \mathord {\left( g + \varphi \right) } \circ K^*\right) }\mathord {\left( y\right) }$.)

Thus, if $\mathord {\left( {\bar{x}}, {\bar{y}}\right) } \in \mathcal {H}\times \mathcal {G}$ is a critical point of the objective function $\Phi$, then ${\bar{x}}$ is a critical point of $g + \varphi - h \circ K$ and ${\bar{y}}$ is a critical point of $h^* - \mathord {\left( g + \varphi \right) }^* \circ K^*$.

## The algorithm

Let $(x_0, y_0) \in \mathcal {H}\times \mathcal {G}$, and let $\mathord {\left( \gamma _n\right) }_{n\ge 0}$ and $\mathord {\left( \mu _n\right) }_{n\ge 0}$ be sequences of positive numbers. We propose the following iterative scheme: For all $n\ge 0$ set

9 $$\begin{aligned} x_{n+1}&= {{\mathrm{Prox}}}_{\gamma _n g} \mathord {\left( x_n + \gamma _n K^* y_n - \gamma _n \nabla \varphi \mathord {\left( x_n\right) }\right) }, \end{aligned}$$

10 $$\begin{aligned} y_{n+1}&= {{\mathrm{Prox}}}_{\mu _n h^*} \mathord {\left( y_n + \mu _n K x_{n+1}\right) }. \end{aligned}$$

By the inequalities for the proximal points, we have, for every $x, y \in \mathcal {H}$ and $n\ge 0$,

$$\begin{aligned} g\mathord {\left( x_{n+1}\right) } - g\mathord {\left( x\right) } \le&\frac{1}{\gamma _n} \left\langle x_n + \gamma _n K^* y_n - \gamma _n \nabla \varphi \mathord {\left( x_n\right) } - x_{n+1}, x_{n+1} - x \right\rangle \\ =&\frac{1}{\gamma _n} \left\langle x_n - x_{n+1}, x_{n+1} - x \right\rangle + \left\langle K^* y_n, x_{n+1} - x \right\rangle \\&- \left\langle \nabla \varphi \mathord {\left( x_n\right) }, x_{n+1} - x \right\rangle , \\ h^*\mathord {\left( y_{n+1}\right) } - h^*\mathord {\left( y\right) } \le&\frac{1}{\mu _n} \left\langle y_n + \mu _n K x_{n+1} - y_{n+1}, y_{n+1} - y \right\rangle \\ =&\frac{1}{\mu _n} \left\langle y_n - y_{n+1}, y_{n+1} - y \right\rangle + \left\langle K x_{n+1}, y_{n+1} - y \right\rangle . \end{aligned}$$

Moreover, using [21, Theorem 18.15 (iii)] and the subdifferential inequality, we have for every $x \in \mathcal {H}$ and $n\ge 0$,

$$\begin{aligned} \varphi \mathord {\left( x_{n+1}\right) } - \varphi \mathord {\left( x_n\right) }&\le \left\langle \nabla \varphi \mathord {\left( x_n\right) }, x_{n+1} - x_n \right\rangle + \frac{1}{2\beta } \left\| x_n - x_{n+1} \right\| ^2, \\ \varphi \mathord {\left( x_n\right) } - \varphi \mathord {\left( x\right) }&\le \left\langle \nabla \varphi \mathord {\left( x_n\right) }, x_n - x \right\rangle . \end{aligned}$$

We consider the auxiliary function $\Phi : \mathcal {H}\times \mathcal {G}\rightarrow \overline{\mathbb {R}}$ defined by

$$\begin{aligned} \Phi \mathord {\left( x, y\right) } = g\mathord {\left( x\right) } + \varphi \mathord {\left( x\right) } + h^*\mathord {\left( y\right) } - \left\langle y, Kx \right\rangle . \end{aligned}$$

By the inequalities above, we have, for arbitrary $x\in \mathcal {H}$, $y \in \mathcal {G}$ and $n\ge 0$,

11 $$\begin{aligned}&\Phi \mathord {\left( x_{n+1}, y_{n+1}\right) } - \Phi \mathord {\left( x, y\right) } \nonumber \\&\quad = g\mathord {\left( x_{n+1}\right) } - g\mathord {\left( x\right) } + \varphi \mathord {\left( x_{n+1}\right) } - \varphi \mathord {\left( x\right) } + h^*\mathord {\left( y_{n+1}\right) } - h^*\mathord {\left( y\right) } \nonumber \\&\qquad + \left\langle y, Kx \right\rangle - \left\langle y_{n+1}, Kx_{n+1} \right\rangle \nonumber \\&\quad \le \frac{1}{\gamma _n} \left\langle x_n - x_{n+1}, x_{n+1} - x \right\rangle + \frac{1}{\mu _n}\left\langle y_n - y_{n+1}, y_{n+1} - y \right\rangle + \frac{1}{2\beta } \left\| x_n - x_{n+1} \right\| ^2 \nonumber \\&\qquad + \left\langle K\mathord {\left( x - x_{n+1}\right) }, y - y_n \right\rangle . \end{aligned}$$

Furthermore, for any $n \ge 0$,

12 $$\begin{aligned} \Phi \mathord {\left( x_{n+1}, y_n\right) } - \Phi \mathord {\left( x_n, y_n\right) }&= g\mathord {\left( x_{n+1}\right) } + \varphi \mathord {\left( x_{n+1}\right) } - g\mathord {\left( x_n\right) } - \varphi \mathord {\left( x_n\right) } \nonumber \\&+\, \left\langle K^* y_n, x_n - x_{n+1} \right\rangle \nonumber \\&\le \,\mathord {\left( \frac{1}{2\beta } - \frac{1}{\gamma _n}\right) } \left\| x_n - x_{n+1} \right\| ^2, \end{aligned}$$

13 $$\begin{aligned} \Phi \mathord {\left( x_{n+1}, y_{n+1}\right) } - \Phi \mathord {\left( x_{n+1}, y_n\right) }&= h^*\mathord {\left( y_{n+1}\right) } - h^*\mathord {\left( y_n\right) } + \left\langle y_n - y_{n+1}, K x_{n+1} \right\rangle \nonumber \\&\le -\frac{1}{\mu _n} \left\| y_n - y_{n+1} \right\| ^2. \end{aligned}$$

The last two inequalities give rise to the following statement.

### Proposition 2

For each $n\ge 0$, we have

$$\begin{aligned} \Phi \mathord {\left( x_{n+1}, y_{n+1}\right) } \le \Phi \mathord {\left( x_{n+1}, y_n\right) } \le \Phi \mathord {\left( x_n, y_n\right) }, \end{aligned}$$

provided that $$0 < \gamma _n \le 2\beta $$.

### Proposition 3

Let

14 $$\begin{aligned} 0< \inf _{n_\ge 0} \gamma _n \le \sup _{n\ge 0} \gamma _n< 2\beta \qquad \text {and} \qquad 0< \inf _{n\ge 0} \mu _n \le \sup _{n\ge 0} \mu _n < +\infty . \end{aligned}$$

Furthermore, let $\inf \left\{ g\mathord {\left( x\right) } + \varphi \mathord {\left( x\right) } - h\mathord {\left( Kx\right) } \,\Big \vert \, x\in \mathcal {H} \right\} > -\infty$. Then,

$$\begin{aligned} \sum _{n \ge 0} \left\| x_n - x_{n+1} \right\| ^2< +\infty \qquad \text {and} \qquad \sum _{n\ge 0} \left\| y_n - y_{n+1} \right\| ^2 < +\infty . \end{aligned}$$

### Proof

Let $N \ge 1$ be an integer. Sum up (12) and (13) for $n = 0, \ldots , N-1$ and obtain

$$\begin{aligned}&\Phi \mathord {\left( x_N, y_N\right) } - \Phi \mathord {\left( x_0, y_0\right) } \le \sum _{n = 0}^{N-1} \mathord {\left( \frac{1}{2\beta } - \frac{1}{\gamma _n}\right) } \left\| x_n - x_{n+1} \right\| ^2 \\&\quad - \sum _{n = 0}^{N-1} \frac{1}{\mu _n} \left\| y_n - y_{n+1} \right\| ^2. \end{aligned}$$

By assumption, the expression on the left-hand side is bounded from below by a fixed real number *M* for any $N \ge 1$, and so is the right-hand side. The numbers $\mathord {\left( \frac{1}{\gamma _n} - \frac{1}{2\beta }\right) }$ and $\frac{1}{\mu _n}$ are bounded from below by a positive number, say $\varepsilon > 0$, so

$$\begin{aligned} \sum _{n = 0}^{N-1} \left\| x_n - x_{n+1} \right\| ^2 + \sum _{n = 0}^{N-1} \left\| y_n - y_{n+1} \right\| ^2 \le -\frac{M}{\varepsilon }. \end{aligned}$$

Since *N* is arbitrary, the series converge. $\square$

### Proposition 4

Let $\inf \left\{ g\mathord {\left( x\right) } + \varphi \mathord {\left( x\right) } - h\mathord {\left( Kx\right) } \,\Big \vert \, x\in \mathcal {H} \right\} > -\infty$ and (14) be satisfied. If $\mathord {\left( x_n\right) }_{n\ge 0}$ and $\mathord {\left( y_n\right) }_{n\ge 0}$ are bounded, then

1. Every cluster point of $\mathord {\left( x_n\right) }_{n\ge 0}$ is a critical point of (2),
2. Every cluster point of $\mathord {\left( y_n\right) }_{n\ge 0}$ is a critical point of (3) and
3. Every cluster point of $\mathord {\left( x_n, y_n\right) }_{n\ge 0}$ is a critical point of (4).

### Proof

Let ${\bar{x}}$ be a cluster point of $\mathord {\left( x_n\right) }_{n\ge 0}$. Let $\mathord {\left( x_{n_k}\right) }_{k\ge 0}$ be a subsequence of $\mathord {\left( x_n\right) }_{n\ge 0}$ such that $x_{n_k} \rightarrow {\bar{x}}$. By another transition to a subsequence, we can guarantee $y_{n_k} \rightarrow {\bar{y}}$ for some ${\bar{y}} \in \mathcal {H}$, since $\mathord {\left( y_{n_k}\right) }_{k\ge 0}$ is bounded. By (9) and (10), we obtain, for every $k\ge 0$,

$$\begin{aligned}&\frac{x_{n_k} - x_{n_k+1}}{\gamma _{n_k}} + K^* y_{n_k} - \nabla \varphi \mathord {\left( x_{n_k}\right) } \in \partial g\mathord {\left( x_{n_k+1}\right) } \\&\quad \text {and } \frac{y_{n_k} - y_{n_k + 1}}{\mu _{n_k}} + K x_{n_k+1} \in \partial h^*\mathord {\left( y_{n_k + 1}\right) }, \end{aligned}$$

respectively. By Proposition 3, the first summands on the left-hand side of the above inclusions tend to zero as $k\rightarrow \infty$. Using the continuity of $\nabla \varphi$ and the closedness of the graphs of $\partial g$ and $\partial h^*$ and passing to the limit, we get $K^* {\bar{y}} - \nabla \varphi \mathord {\left( {\bar{x}}\right) } \in \partial g\mathord {\left( {\bar{x}}\right) }$ and $K {\bar{x}} \in \partial h^*\mathord {\left( {\bar{y}}\right) }$, which means that $\mathord {\left( {\bar{x}}, {\bar{y}}\right) }$ is a critical point of $\Phi$. The first statement follows by considering Remark 1. For the second statement, one has to choose ${\bar{x}}$ and ${\bar{y}}$ in reverse order, for the third one, they are chosen at the same time. $\square$

### Remark 2

It is clear that one cannot expect the cluster points to be minima, since it is easy to see that $\mathord {\left( {\bar{x}}, {\bar{y}}\right) }$ is a fixed point of the iteration (9)–(10) if and only if (5) and (6) are satisfied, i.e., if and only if $\mathord {\left( {\bar{x}}, {\bar{y}}\right) }$ is a critical point for $\Phi$ (independent of the choice of the parameters $\mathord {\left( \gamma _n\right) }_{n\ge 0}$ and $\mathord {\left( \mu _n\right) }_{n\ge 0}$).

### Remark 3

One should notice that the iterative scheme given by (9) and (10) does not use any subgradients, but only proxmial points and gradients, which are continuous with respect to the input. In contrast, the DCA and its variants use the subgradients of at least one of the involved functions. The performance might therefore depend on the subgradient oracle, whereas our algorithm is determined by the choice of the starting points and the stepsize sequences alone. This is especially an issue when dealing with nonsmooth functions like the $\ell ^1$ norm.

### Example 1

**Fig. 1:**
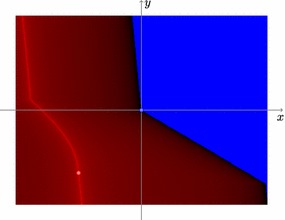
Regions of convergence for Example 1. The horizontal and vertical axes indicate the primal and dual starting points, respectively. For points in the blue region, the method converges to the stationary point $\mathord {\left( 0, 0\right) }$, which is not a local minimum, and for points in the red region it converges to the global minimum $\mathord {\left( -1, -1\right) }$. The stationary points are highlighted with circles. Light colours mean fewer iterations for convergence than darker colours. The stepsize sequences are chosen to be constant: $\gamma _n = \mu _n = 0.1$ for all $n\ge 0$

Consider the problem given in [24, Example 4]. The primal problem

$$\begin{aligned} \min _{x\in \mathbb {R}} \left\{ \frac{1}{2} x^2 - \max \left\{ -x, 0 \right\} \right\} \end{aligned}$$

and its Toland dual

$$\begin{aligned} \min _{y\in \left[ -1, 0\right] } \left\{ -\frac{1}{2} y^2 \right\} \end{aligned}$$

have two primal-dual stationary points $\mathord {\left( x, y\right) } = \mathord {\left( 0, 0\right) }$ and $\mathord {\left( x, y\right) } = \mathord {\left( -1, -1\right) }$, but only the latter is a local minimum for any of these problems. On the other hand, the classical DCA might converge to the former stationary point for an unfavourable choice of the subgradients. The same might happen to the double-proximal d.c. algorithm, see Fig. 1. A possible solution to the problem of getting stuck in stationary points which are not local minima is the use of inertial techniques according to Polyak [25], which are already well-established in proximal algorithms for convex and nonconvex problems, see e.g. [26, 27].

### Proposition 5

Let (14) be satisfied. For any $n\ge 0$, the following statements are equivalent:

1. $\mathord {\left( x_n, y_n\right) }$ is a critical point of $\Phi$;
2. $$\mathord {\left( x_{n+1}, y_{n+1}\right) } = \mathord {\left( x_n, y_n\right) }$$;
3. $$\Phi \mathord {\left( x_{n+1}, y_{n+1}\right) } = \Phi \mathord {\left( x_n, y_n\right) }$$.

### Proof

It is easily seen by the formula (1) that the first two statements are equivalent. The equivalence of the latter two follows by (12) and (13). $\square$

Next, we summarise the convergence properties of the prox-prox algorithm. To this end, we denote by $\omega \mathord {\left( x_0, y_0\right) }$ the set of cluster points of the iteration generated by (9) and (10) with the initial points $x_0$ and $y_0$. See also [18, Lemma 5] for an analogous result for a nonconvex forward-backward scheme.

### Lemma 1

Let $\mathcal {H}$ and $\mathcal {G}$ be two real finite-dimensional Hilbert spaces, let $g: \mathcal {H}\rightarrow \overline{\mathbb {R}}$ and $h: \mathcal {G}\rightarrow \overline{\mathbb {R}}$ be proper, convex, and lower semicontinuous functions. Let $\varphi : \mathcal {H}\rightarrow \mathbb {R}$ be a convex, Fréchet differentiable function with a $\frac{1}{\beta }$-Lipschitz continuous gradient, for some $\beta >0$, and let $K: \mathcal {H}\rightarrow \mathcal {G}$ be a linear mapping. Let the sequences $\mathord {\left( \gamma _n\right) }_{n\ge 0}$ and $\mathord {\left( \mu _n\right) }_{n\ge 0}$ satisfy (14). Moreover, assume that the sequence $\mathord {\left( x_n, y_n\right) }_{n \ge 0}$ generated by (9) and (10) is bounded. Then the following assertions hold:

1. $$\emptyset \ne \omega \mathord {\left( x_0, y_0\right) } \subseteq \mathrm {crit}\Phi \subseteq \mathrm {crit}\mathord {\left( g + \varphi - h \circ K\right) } \times \mathrm {crit}\mathord {\left( h^* - \mathord {\left( g + \varphi \right) }^* \circ K^*\right) }$$,
2. $$\lim _{n \rightarrow \infty } \mathrm {dist}\mathord {\left( \mathord {\left( x_n, y_n\right) }, \omega \mathord {\left( x_0, y_0\right) }\right) } = 0$$,
3. if the common optimal value of the problems (2), (3), and (4) is $> -\infty$, then $\omega \mathord {\left( x_0, y_0\right) }$ is a nonempty, compact, and connected set, and so are the sets of the limit points of the sequences $\mathord {\left( x_n\right) }_{n\ge 0}$ and $\mathord {\left( y_n\right) }_{n\ge 0}$,
4. the objective function $\Phi$ is finite and constant on $\omega \mathord {\left( x_0, y_0\right) }$ provided that the optimal value is finite.

### Proof

1. It is clear that the set of cluster points of a bounded sequence is nonempty. That every cluster point is critical for $\Phi$, is the statement of Proposition 4. The last inclusion is discussed in Remark 1.
2. Assume that the assertion does not hold. In this case, there exists an $\varepsilon > 0$ and a subsequence $\mathord {\left( x_{n_k}, y_{n_k}\right) }_{k\ge 0}$ of $\mathord {\left( x_n, y_n\right) }_{n\ge 0}$ with $\mathrm {dist}\mathord {\left( \mathord {\left( x_{n_k}, y_{n_k}\right) }, \omega \mathord {\left( x_0, y_0\right) }\right) } > \varepsilon$ for all $k\ge 0$. The subsequence is bounded, so it has a cluster point, which is a cluster point of the original sequence $\mathord {\left( x_n, y_n\right) }_{n\ge 0}$ as well, thus an element of $\omega \mathord {\left( x_0, y_0\right) }$. This contradicts the assumption $\mathrm {dist}\mathord {\left( \mathord {\left( x_{n_k}, y_{n_k}\right) }, \omega \mathord {\left( x_0, y_0\right) }\right) } > \varepsilon$ for all $k\ge 0$.
3. Since the sequence $\mathord {\left( x_n, y_n\right) }_{n\ge 0}$ is bounded, the sets
  $$\begin{aligned} \Omega _k \mathrel {\mathop :}={{\mathrm{cl}}}\mathord {\left( \bigcup _{n\ge k} \left\{ \mathord {\left( x_n, y_n\right) } \right\} \right) } \end{aligned}$$
  are bounded and closed, hence compact for any $k\ge 0$. Their intersection $\bigcap _{n\ge 0} \Omega _n$, which equals the set of cluster points of $\mathord {\left( x_n, y_n\right) }_{n\ge 0}$, is therefore compact, too. The connectedness follows from the property given by Proposition 3, and the proof is completely analogous to the one of [18, Lemma 5 (iii)].
4. According to Proposition 2, the function values $\Phi \mathord {\left( x_n, y_n\right) }$ are monotonically decreasing, thus convergent, say $\Phi \mathord {\left( x_n, y_n\right) } \rightarrow \ell$. Let $\mathord {\left( {\bar{x}}, {\bar{y}}\right) }$ be an arbitrary limit point of the sequence $\mathord {\left( x_n, y_n\right) }_{n\ge 0}$, and let $\mathord {\left( x_{n_k}, y_{n_k}\right) }_{k\ge 0}$ be a subsequence converging to $\mathord {\left( {\bar{x}}, {\bar{y}}\right) }$ as $k\rightarrow \infty$. By lower semicontinuity, we have $\Phi \mathord {\left( {\bar{x}}, {\bar{y}}\right) } \le \lim _{k\rightarrow \infty } \Phi \mathord {\left( x_{n_k}, y_{n_k}\right) } = \ell$. On the other hand, consider (11) with $x = {\bar{x}}$ and $y = {\bar{y}}$. The right-hand side converges to 0 as we let $n\rightarrow \infty$ along the subsequence $\mathord {\left( n_k\right) }_{k \ge 0}$, so $\ell = \lim _{n\rightarrow \infty } \Phi \mathord {\left( x_n, y_n\right) } \le \Phi \mathord {\left( {\bar{x}}, {\bar{y}}\right) }$. $\square$

### Remark 4

To guarantee the boundedness of the iterates, one could assume that the objective function of the primal-dual minimisation problem (4) is coercive, i.e., the lower level sets are bounded.

## Convergence under Kurdyka–Łojasiewicz assumptions

In the next step, we shall assume the Kurdyka–Łojasiewicz property for the functions involved. Let us recall the definition and some basic properties. By $\Theta _\eta$, for $\eta \in \left( 0, +\infty \right]$, we denote the set of all concave and continuous functions $\vartheta : \left[ 0, \eta \right) \rightarrow \mathbb {R}$ with the following properties:

1. $$\vartheta \mathord {\left( 0\right) } = 0$$,
2. $\vartheta$ is continuously differentiable on $\left( 0, \eta \right)$ and continuous at 0,
3. $$\vartheta '\mathord {\left( s\right) } > 0$$ for all $$s\in \left( 0, \eta \right) $$.

### Definition 2

Let $\mathcal {H}$ be a real finite-dimensional Hilbert space, and let $\Phi : \mathcal {H}\rightarrow \overline{\mathbb {R}}$ be a proper and lower semicontinuous function. We say that $\Phi$ satisfies the *Kurdyka–Łojasiewicz property* at ${\bar{x}} \in {{\mathrm{dom}}}~ \partial _L \Phi \mathrel {\mathop :}=\left\{ x\in \mathcal {H} \,\Big \vert \, \partial _L \Phi \mathord {\left( x\right) } \ne \emptyset \right\}$ if there exists some $\eta \in \left( 0, +\infty \right]$, a neighbourhood *U* of ${\bar{x}}$ and a function $\vartheta \in \Theta _\eta$ such that for all

$$\begin{aligned} x \in U \cap \left\{ x\in \mathcal {H} \,\Big \vert \, \Phi \mathord {\left( {\bar{x}}\right) }< \Phi \mathord {\left( x\right) } < \Phi \mathord {\left( {\bar{x}}\right) } + \eta \right\} \end{aligned}$$

the following inequality holds:

$$\begin{aligned} \vartheta '\mathord {\left( \Phi \mathord {\left( x\right) } - \Phi \mathord {\left( {\bar{x}}\right) }\right) } \cdot \mathrm {dist}\mathord {\left( 0, \partial _L \Phi \mathord {\left( x\right) }\right) } \ge 1. \end{aligned}$$

We call $\Phi$ a *KŁ function* if it satisfies the Kurdyka–Łojasiewicz property at each point ${\bar{x}} \in {{\mathrm{dom}}}~ \partial _L \Phi$.

The following uniform KŁ property is according to [18, Lemma 6].

### Lemma 2

Let $\Omega$ be a compact set, and let $\Phi : \mathcal {H}\rightarrow \overline{\mathbb {R}}$ be a proper and lower semicontinuous function. Assume that $\Phi$ is constant on $\Omega$ and satisfies the KŁ property at each point of $\Omega$. Then there exist $\varepsilon > 0$, $\eta > 0$, and $\vartheta \in \Theta _\eta$ such that for all ${\bar{u}} \in \Omega$ and all *u* in the intersection

15 $$\begin{aligned} \left\{ u \in \mathcal {H} \,\Big \vert \, \mathrm {dist}\mathord {\left( u, \Omega \right) }< \varepsilon \right\} \cap \left\{ u\in \mathcal {H} \,\Big \vert \, \Phi \mathord {\left( {\bar{u}}\right) }< \Phi \mathord {\left( u\right) } < \Phi \mathord {\left( {\bar{u}}\right) } + \eta \right\} \end{aligned}$$

one has

$$\begin{aligned} \vartheta '\mathord {\left( \Phi \mathord {\left( u\right) } - \Phi \mathord {\left( {\bar{u}}\right) }\right) } \cdot \mathrm {dist}\mathord {\left( 0, \partial _L \Phi \mathord {\left( u\right) }\right) } \ge 1. \end{aligned}$$

In the KŁ property, we need the distance of a subgradient from zero. In our algorithm, we have the following result.

### Lemma 3

For each $n\ge 1$ with $\gamma _{n-1} < 2\beta$, there exist $\mathord {\left( x^*_n, y^*_n\right) } \in \mathcal {H}\times \mathcal {G}$ with $\mathord {\left( x^*_n, y^*_n\right) } \in \partial _L \Phi \mathord {\left( x_n, y_n\right) }$ and

$$\begin{aligned} \left\| x^*_n \right\|&\le \left\| K \right\| \left\| y_{n-1} - y_n \right\| + \frac{1}{\gamma _{n-1}} \left\| x_{n-1} - x_n \right\| , \\ \left\| y^*_n \right\|&\le \frac{1}{\mu _{n-1}} \left\| y_{n-1} - y_n \right\| . \end{aligned}$$

### Proof

From the definition of the algorithm, we have, for each $n \ge 1$,

$$\begin{aligned}&\frac{x_{n-1} - x_n}{\gamma _{n-1}} + K^* y_{n-1} - \nabla \varphi \mathord {\left( x_{n-1}\right) } \in \partial g\mathord {\left( x_n\right) }, \\&\frac{y_{n-1} - y_n}{\mu _{n-1}} + K x_n \in \partial h^*\mathord {\left( y_n\right) }. \end{aligned}$$

Consider the function ${\widetilde{\Phi }}: \mathcal {H}\times \mathcal {G}\rightarrow \overline{\mathbb {R}}, {\widetilde{\Phi }}\mathord {\left( x, y\right) } \mathrel {\mathop :}=g\mathord {\left( x\right) } + \varphi \mathord {\left( x\right) } + h^*\mathord {\left( y\right) }$. By the usual calculus of the convex subdifferential and [28, Proposition 8.12], for each $n \ge 1$

$$\begin{aligned} \partial _L {\widetilde{\Phi }}\mathord {\left( x_n, y_n\right) } = \mathord {\left( \partial g\mathord {\left( x_n\right) } + \nabla \varphi \mathord {\left( x_n\right) }\right) } \times \partial h^*\mathord {\left( y_n\right) }. \end{aligned}$$

By [28, Exercise 8.8], we have for each $n \ge 1$

16 $$\begin{aligned} \partial _L \Phi \mathord {\left( x_n, y_n\right) }&= \partial _L {\widetilde{\Phi }}\mathord {\left( x_n, y_n\right) } - \mathord {\left( K^* y_n, Kx_n\right) } \nonumber \\&= \mathord {\left( \partial g\mathord {\left( x_n\right) } + \nabla \varphi \mathord {\left( x_n\right) } - K^* y_n\right) } \times \mathord {\left( \partial h^*\mathord {\left( y_n\right) } - Kx_n\right) }, \end{aligned}$$

thus,

$$\begin{aligned} \begin{pmatrix} x_n^* \\ y_n^* \end{pmatrix} \mathrel {\mathop :}=\begin{pmatrix} \frac{x_{n-1} - x_n}{\gamma _{n-1}} + \nabla \varphi \mathord {\left( x_n\right) } - \nabla \varphi \mathord {\left( x_{n-1}\right) } + K^*\mathord {\left( y_{n-1} - y_n\right) } \\ \frac{y_{n-1} - y_n}{\mu _{n-1}} \end{pmatrix}\in \partial _L \Phi \mathord {\left( x_n, y_n\right) }. \end{aligned}$$

Now, we estimate for each $n \ge 1$

$$\begin{aligned} \left\| x_n^* \right\|&\le \left\| K \right\| \left\| y_{n-1} - y_n \right\| + \frac{1}{\gamma _{n-1}}\left\| \mathord {\left( \mathrm {Id}- \gamma _{n-1} \nabla \varphi \right) }\mathord {\left( x_{n-1}\right) } - \mathord {\left( \mathrm {Id}- \gamma _{n-1} \nabla \varphi \right) }\mathord {\left( x_n\right) } \right\| . \end{aligned}$$

By the Baillon–Haddad theorem [21, Corollary 18.16], $\nabla \varphi$ is $\beta$-cocoercive. By [21, Proposition 4.33], $\mathrm {Id}- \gamma _{n-1} \nabla \varphi$ is nonexpansive for $\gamma _{n-1} < 2\beta$, which leads to the desired conclusion. $\square$

### The case when $\Phi$ is a KŁ function

#### Theorem 1

Let

$$\begin{aligned} 0< \underline{\gamma }\mathrel {\mathop :}=\inf _{n\ge 0} \gamma _n&\le \overline{\gamma }\mathrel {\mathop :}=\sup _{n\ge 0} \gamma _n< \beta , \\ 0< \underline{\mu }\mathrel {\mathop :}=\inf _{n\ge 0} \mu _n&\le \overline{\mu }\mathrel {\mathop :}=\sup _{n\ge 0} \mu _n < +\infty . \end{aligned}$$

Suppose that $\Phi$ is in addition a KŁ function bounded from below. Then $\mathord {\left( x_n, y_n\right) }_{n\ge 0}$ is a Cauchy sequence, thus convergent to a critical point of $\Phi$.

#### Proof

Let $\Omega \mathrel {\mathop :}=\omega \mathord {\left( x_0, y_0\right) }$, and let $\ell \in \mathbb {R}$ be the value of $\Phi$ on $\Omega$ (see item 1 of Lemma 1). If $\Phi \mathord {\left( x_n, y_n\right) } = \ell$ for some $n \ge 0$, then, by (12) and (13), $x_{n+1} = x_n$ and $y_{n+1} = y_n$, and the assertion holds. Therefore, we assume $\Phi \mathord {\left( x_n, y_n\right) } > \ell$ for all $n\ge 0$.

Let $\varepsilon > 0$, $\eta > 0$ and $\vartheta \in \Theta _\eta$ be as provided by Lemma 2. Since $\Phi \mathord {\left( x_n, y_n\right) } \rightarrow \ell$ as $n\rightarrow +\infty$, we find $n_1 \ge 0$ with $\Phi \mathord {\left( x_n, y_n\right) } < \ell + \eta$ for $n\ge n_1$. Since $\mathrm {dist}\mathord {\left( \mathord {\left( x_n, y_n\right) }, \Omega \right) } \rightarrow 0$ as $n\rightarrow +\infty$, we find $n_2 \ge 0$ with $\mathrm {dist}\mathord {\left( \mathord {\left( x_n, y_n\right) }, \Omega \right) } < \varepsilon$ for $n\ge n_2$.

In the following, fix an arbitrary $n\ge n_0 \mathrel {\mathop :}=\max \left\{ n_1, n_2, 1 \right\}$. Then $\mathord {\left( x_n, y_n\right) }$ is an element of the intersection (15). Consequently,

17 $$\begin{aligned} \vartheta '\mathord {\left( \Phi \mathord {\left( x_n, y_n\right) } - \Phi \mathord {\left( {\bar{x}}, {\bar{y}}\right) }\right) } \cdot \mathrm {dist}\mathord {\left( \mathord {\left( 0, 0\right) }, \partial _L \Phi \mathord {\left( x_n, y_n\right) }\right) } \ge 1. \end{aligned}$$

By the concavity of $\vartheta$, we get, for all $s \in \mathord {\left( 0, \eta \right) }$,

$$\begin{aligned}&\vartheta \mathord {\left( s\right) } - \vartheta \mathord {\left( \Phi \mathord {\left( x_n, y_n\right) } - \Phi \mathord {\left( {\bar{x}}, {\bar{y}}\right) }\right) } \\&\quad \le \vartheta '\mathord {\left( \Phi \mathord {\left( x_n, y_n\right) } - \Phi \mathord {\left( {\bar{x}}, {\bar{y}}\right) }\right) } \cdot \mathord {\left( s - \Phi \mathord {\left( x_n, y_n\right) } + \Phi \mathord {\left( {\bar{x}}, {\bar{y}}\right) }\right) }, \end{aligned}$$

so, setting in particular $s := \Phi \mathord {\left( x_{n+1}, y_{n+1}\right) } - \Phi \mathord {\left( {\bar{x}}, {\bar{y}}\right) } \in \left( 0, \eta \right)$,

$$\begin{aligned}&\mathord {\left( \vartheta \mathord {\left( \Phi \mathord {\left( x_n, y_n\right) } - \Phi \mathord {\left( {\bar{x}}, {\bar{y}}\right) }\right) } - \vartheta \mathord {\left( \Phi \mathord {\left( x_{n+1}, y_{n+1}\right) } - \Phi \mathord {\left( {\bar{x}}, {\bar{y}}\right) }\right) }\right) } \cdot \left\| \mathord {\left( x_n^*, y_n^*\right) } \right\| \\&\quad \ge \vartheta '\mathord {\left( \Phi \mathord {\left( x_n, y_n\right) } - \Phi \mathord {\left( {\bar{x}}, {\bar{y}}\right) }\right) } \cdot \mathord {\left( \Phi \mathord {\left( x_n, y_n\right) } - \Phi \mathord {\left( x_{n+1}, y_{n+1}\right) }\right) } \cdot \left\| \mathord {\left( x_n^*, y_n^*\right) } \right\| \\&\quad \ge \vartheta '\mathord {\left( \Phi \mathord {\left( x_n, y_n\right) } - \Phi \mathord {\left( {\bar{x}}, {\bar{y}}\right) }\right) } \cdot \mathord {\left( \Phi \mathord {\left( x_n, y_n\right) } - \Phi \mathord {\left( x_{n+1}, y_{n+1}\right) }\right) } \cdot \mathrm {dist}\mathord {\left( \mathord {\left( 0, 0\right) }, \partial _L \Phi \mathord {\left( x_n, y_n\right) }\right) } \\&\quad \ge \mathord {\left( \Phi \mathord {\left( x_n, y_n\right) } - \Phi \mathord {\left( x_{n+1}, y_{n+1}\right) }\right) }. \end{aligned}$$

Moreover, by (12) and (13),

$$\begin{aligned} \Phi \mathord {\left( x_n, y_n\right) } - \Phi \mathord {\left( x_{n+1}, y_{n+1}\right) } \ge \mathord {\left( \frac{1}{\gamma _n} - \frac{1}{2\beta }\right) } \left\| x_n - x_{n+1} \right\| ^2 + \frac{1}{\mu _n} \left\| y_n - y_{n+1} \right\| ^2. \end{aligned}$$

Let us define the following shorthands:

$$\begin{aligned} \delta _n&\mathrel {\mathop :}=\sqrt{\mathord {\left( \frac{1}{\gamma _n} - \frac{1}{2\beta }\right) } \left\| x_n - x_{n+1} \right\| ^2 + \frac{1}{\mu _n} \left\| y_n - y_{n+1} \right\| ^2}, \\ \varepsilon _n&\mathrel {\mathop :}=\vartheta \mathord {\left( \Phi \mathord {\left( x_n, y_n\right) } - \Phi \mathord {\left( {\bar{x}}, {\bar{y}}\right) }\right) } \end{aligned}$$

for $n\ge n_0$ to obtain the inequality

$$\begin{aligned} \mathord {\left( \varepsilon _n - \varepsilon _{n+1}\right) } \cdot \left\| \mathord {\left( x_n^*, y_n^*\right) } \right\| \ge \delta _n^2. \end{aligned}$$

By the arithmetic-geometric inequality, for any $r > 0$ and $n\ge n_0$

18 $$\begin{aligned} \delta _n&\le \sqrt{\mathord {\left( r\left\| \mathord {\left( x_n^*, y_n^*\right) } \right\| \right) } \cdot \mathord {\left( \frac{1}{r}\mathord {\left( \varepsilon _n - \varepsilon _{n+1}\right) }\right) }} \nonumber \\&\le \frac{1}{2} \mathord {\left( r\left\| \mathord {\left( x_n^*, y_n^*\right) } \right\| + \frac{1}{r} \mathord {\left( \varepsilon _n - \varepsilon _{n+1}\right) }\right) } \nonumber \\&\le r\left\| \mathord {\left( x_n^*, y_n^*\right) } \right\| + \frac{1}{r} \mathord {\left( \varepsilon _n - \varepsilon _{n+1}\right) } \end{aligned}$$

(recall that, by Proposition 2 and the properties of $\vartheta$, the sequence $\mathord {\left( \varepsilon _n\right) }_{n\ge n_0}$ is decreasing, so $\varepsilon _n - \varepsilon _{n+1} \ge 0$). On the other hand, by Lemma 3 and the inequality $2ab \le a^2 + b^2$ ($a, b \ge 0$), for any $n\ge n_0$

19 $$\begin{aligned} \left\| \mathord {\left( x_n^*, y_n^*\right) } \right\| ^2&\le \mathord {\left( \left\| K \right\| ^2 + \frac{1}{\mu _{n-1}^2}\right) } \left\| y_{n-1} - y_n \right\| ^2 + \frac{1}{\gamma _{n-1}^2} \left\| x_{n-1} - x_n \right\| ^2 \nonumber \\&\quad + \frac{2\left\| K \right\| }{\gamma _{n-1}} \left\| x_{n-1} - x_n \right\| \left\| y_{n-1} - y_n \right\| \nonumber \\&\le \mathord {\left( 2\left\| K \right\| ^2 + \frac{1}{\mu _{n-1}^2}\right) } \left\| y_{n-1} - y_n \right\| ^2 + \frac{2}{\gamma _{n-1}^2} \left\| x_{n-1} - x_n \right\| ^2 \nonumber \\&\le C^2_n \delta ^2_{n-1}, \end{aligned}$$

with

$$\begin{aligned} C_n \mathrel {\mathop :}=&\sqrt{\max \left\{ \frac{\frac{2}{\gamma _{n-1}^2}}{\frac{1}{\gamma _{n-1}} - \frac{1}{2\beta }}, \frac{2\left\| K \right\| ^2 + \frac{1}{\mu _{n-1}^2}}{\frac{1}{\mu _{n-1}}} \right\} }\\ =&\sqrt{\max \left\{ \frac{4\beta }{\gamma _{n-1}\mathord {\left( 2\beta - \gamma _{n-1}\right) }}, \frac{1 + 2\left\| K \right\| ^2 \mu _{n-1}^2}{\mu _{n-1}} \right\} }. \end{aligned}$$

For all $$n\ge n_0$$,

$$\begin{aligned} C_n \le C_0 \mathrel {\mathop :}=\sqrt{\max \left\{ \frac{4\beta }{\underline{\gamma }\mathord {\left( 2\beta - \overline{\gamma }\right) }}, \frac{1 + 2\left\| K \right\| ^2 \overline{\mu }^2}{\underline{\mu }} \right\} }. \end{aligned}$$

Combined with (18), we obtain

20 $$\begin{aligned} \delta _n \le rC_0 \delta _{n-1} + \frac{1}{r} \mathord {\left( \varepsilon _n - \varepsilon _{n+1}\right) }. \end{aligned}$$

For any $k \ge n_0+1$, we have, by iteration,

$$\begin{aligned} \delta _k \le \mathord {\left( rC_0\right) }^{k - n_0} \delta _{n_0} + \sum _{n = 0}^{k - n_0 - 1} \frac{\mathord {\left( rC_0\right) }^n}{r} \mathord {\left( \varepsilon _{k - n} - \varepsilon _{k - n + 1}\right) }, \end{aligned}$$

therefore, for any $N \ge n_0+1$ and $0< r < \frac{1}{C_0}$,

$$\begin{aligned} \sum _{k = n_0+1}^N \delta _k&\le \sum _{k = n_0+1}^N \mathord {\left( \mathord {\left( rC_0\right) }^{k - n_0} \delta _{n_0} + \sum _{n = 0}^{k - n_0 - 1} \frac{\mathord {\left( rC_0\right) }^n}{r} \mathord {\left( \varepsilon _{k - n} - \varepsilon _{k - n + 1}\right) }\right) } \\&= \sum _{k = 0}^{N - n_0-1} \mathord {\left( rC_0\right) }^{k+1} \delta _{n_0} + \sum _{k = 0}^{N - n_0-1} \sum _{n = 0}^{k} \frac{\mathord {\left( rC_0\right) }^n}{r} \mathord {\left( \varepsilon _{k + n_0 - n+1} - \varepsilon _{k + n_0 - n + 2}\right) } \\&\le \frac{rC_0\delta _{n_0}}{1 - rC_0} + \sum _{n = 0}^{N - n_0 - 1} \frac{\mathord {\left( rC_0\right) }^n}{r} \sum _{k = n}^{N - n_0-1} \mathord {\left( \varepsilon _{k + n_0 - n+1} - \varepsilon _{k + n_0 - n + 2}\right) } \\&\le \frac{rC_0\delta _{n_0}}{1 - rC_0} + \sum _{n = 0}^{N - n_0 - 1} \frac{\mathord {\left( rC_0\right) }^n}{r} \varepsilon _{n_0 + 1} \\&\le \frac{rC_0\delta _{n_0}}{1 - rC_0} + \frac{\varepsilon _{n_0 + 1}}{r\mathord {\left( 1 - rC_0\right) }}. \end{aligned}$$

The last right-hand side does not depend on *N*, thus, we conclude that $\sum _{k = n_0+1}^\infty \delta _k$ is finite, and so are $\sum _{k = n_0+1}^\infty \left\| x_n - x_{n+1} \right\|$ and $\sum _{k = n_0+1}^{\infty } \left\| y_n - y_{n+1} \right\|$. $\square$

### Convergence rates

#### Lemma 4

Assume that $\Phi$ is a KŁ function with $\vartheta \mathord {\left( t\right) } = Mt^{1 - \theta }$ for some $M > 0$ and $0 \le \theta < 1$. Let ${\bar{x}}$ and ${\bar{y}}$ the limit points of the sequences $\mathord {\left( x_n\right) }_{n\ge 0}$ and $\mathord {\left( y_n\right) }_{n\ge 0}$, respectively (which exist due to Theorem 1). Then the following convergence rates are guaranteed:

1. If $\theta = 0$, then there exists $n_0 \ge 0$, such that $x_n = x_{n_0}$ and $y_n = y_{n_0}$ for $n\ge n_0$;
2. If $0 < \theta \le \frac{1}{2}$, then there exist $c > 0$ and $0 \le q < 1$ such that
  $$\begin{aligned} \left\| x_n - {\bar{x}} \right\| \le cq^n \quad \text {and}\quad \left\| y_n - {\bar{y}} \right\| \le cq^n \end{aligned}$$
  for all $$n \ge 0$$;
3. If $\frac{1}{2}< \theta < 1$, then there exists $c > 0$ such that
  $$\begin{aligned} \left\| x_n - {\bar{x}} \right\| \le c n^{-\frac{1-\theta }{2\theta - 1}} \quad \text {and}\quad \left\| y_n - {\bar{y}} \right\| \le c n^{-\frac{1 - \theta }{2\theta - 1}} \end{aligned}$$
  for all $$n \ge 0$$.

#### Proof

1. First, let $\theta = 0$. Assume to the contrary (see Proposition 5) that for any $n \ge 0$, $\mathord {\left( x_{n+1}, y_{n+1}\right) } \ne \mathord {\left( x_n, y_n\right) }$. We have $\vartheta '\mathord {\left( t\right) } = M$ for all $t > 0$ and thus, by (17),
  $$\begin{aligned} M \cdot \left\| \mathord {\left( x_n^*, y_n^*\right) } \right\| \ge 1 \ \quad \text{ for } \text{ any } \ n \ge 1, \end{aligned}$$
  which contradicts either Lemma 3 or Proposition 3.

Before considering the other cases, assume from now on that $\mathord {\left( x_n, y_n\right) }$ is not a critical point of $\Phi$ for any $n\ge 0$. Notice that $\vartheta '\mathord {\left( t\right) } = \mathord {\left( 1 - \theta \right) } M t^{-\theta }$. In the proof of Theorem 1, we have shown that for $0<r<\frac{1}{C_0}$

$$\begin{aligned} \sum _{k = n_0+1}^\infty \delta _k&\le \frac{rC_0\delta _{n_0}}{1 - rC_0} + \frac{\varepsilon _{n_0 + 1}}{r\mathord {\left( 1 - rC_0\right) }} \\&= \frac{rC_0\delta _{n_0}}{1 - rC_0} + \frac{M\mathord {\left( \Phi \mathord {\left( x_{n_0 + 1}, y_{n_0 + 1}\right) } - \Phi \mathord {\left( {\bar{x}}, {\bar{y}}\right) }\right) }^{1 - \theta }}{r\mathord {\left( 1 - rC_0\right) }} \\&= \frac{rC_0\delta _{n_0}}{1 - rC_0} + \frac{M^{1 + \frac{1 - \theta }{\theta }}\mathord {\left( 1 - \theta \right) }^{\frac{1 - \theta }{\theta }}}{r\mathord {\left( 1 - rC_0\right) }\vartheta '\mathord {\left( \Phi \mathord {\left( x_{n_0 + 1}, y_{n_0 + 1}\right) } - \Phi \mathord {\left( {\bar{x}}, {\bar{y}}\right) }\right) }^{\frac{1-\theta }{\theta }}} \\&\le \frac{rC_0\delta _{n_0}}{1 - rC_0} + \frac{M^{\frac{1}{\theta }}\mathord {\left( 1 - \theta \right) }^{\frac{1 - \theta }{\theta }}\left\| \mathord {\left( x_{n_0 + 1}^*, y_{n_0 + 1}^*\right) } \right\| ^{\frac{1-\theta }{\theta }}}{r\mathord {\left( 1 - rC_0\right) }}, \end{aligned}$$

where the last inequality follows from the KŁ property (notice that $\Phi \mathord {\left( x_{n_0 + 1}, y_{n_0 + 1}\right) } - \Phi \mathord {\left( {\bar{x}}, {\bar{y}}\right) } > 0$ because we assumed that $\mathord {\left( x_{n_0 + 1}, y_{n_0 + 1}\right) }$ is not a critical point of $\Phi$). We can repeat this calculation for any $n\ge n_0+1$ instead of $n_0+1$, because such an *n* would meet the criteria according to which we chose $n_0+1$. Thus, we obtain from (19), for $n\ge n_0+1$,

21 $$\begin{aligned} \sum _{k = n + 1}^\infty \delta _k \le \frac{rC_0\delta _n}{1 - rC_0} + \frac{M^{\frac{1}{\theta }} \mathord {\left( 1 - \theta \right) }^{\frac{1 - \theta }{\theta }} \mathord {\left( C_0 \delta _n\right) }^{\frac{1 - \theta }{\theta }}}{r\mathord {\left( 1 - rC_0\right) }}. \end{aligned}$$

The rest of the proof follows in the lines of [29, Theorem 2]:

2. Let $0 < \theta \le \frac{1}{2}$. Then $1 \le \frac{1 - \theta }{\theta } < +\infty$, so $\delta _n \rightarrow 0$ as $n\rightarrow \infty$ implies that the first term on the right-hand side of (21) is the dominant one. Therefore, we find $n_1 \ge n_0+1$ and $C_1 > 0$ such that
  $$\begin{aligned} \sum _{k = n + 1}^{\infty } \delta _k \le C_1 \delta _n = C_1 \mathord {\left( \sum _{k = n}^{\infty } \delta _k - \sum _{k = n + 1}^\infty \delta _k\right) } \end{aligned}$$
  for any $n\ge n_1$. Thus, for any $n\ge n_1$,
  $$\begin{aligned} \sum _{k = n + 1}^{\infty } \delta _k \le \frac{C_1}{1 + C_1} \sum _{k = n}^{\infty } \delta _k. \end{aligned}$$
  By induction, for any $n\ge n_1+1$,
  $$\begin{aligned} \delta _{n} \le \sum _{k = n}^\infty \delta _k \le \mathord {\left( \frac{C_1}{1 + C_1}\right) }^{n - n_1} \sum _{k = n_1}^\infty \delta _k, \end{aligned}$$
  which proves the assertion.
3. Let $\frac{1}{2}< \theta < 1$. Then $0< \frac{1 - \theta }{\theta } < 1$, so $\delta _n \rightarrow 0$ as $n\rightarrow \infty$ implies that the second term on the right-hand side of (21) is the dominant one. Therefore, we find $n_1 \ge n_0+1$ and $C_1 > 0$ such that
  $$\begin{aligned} \sum _{k = n+1}^\infty \delta _k \le C_1 \delta _n^{\frac{1 - \theta }{\theta }} \end{aligned}$$
  for any $n\ge n_1$. Then, for any $n\ge n_1$,
  $$\begin{aligned} \mathord {\left( \sum _{k = n+1}^\infty \delta _k\right) }^{\frac{\theta }{1 - \theta }} \le C_1^{\frac{\theta }{1 - \theta }} \mathord {\left( \sum _{k = n}^\infty \delta _k - \sum _{k = n+1}^\infty \delta _k\right) }. \end{aligned}$$
  We define $h: \left( 0, +\infty \right) \rightarrow \mathbb {R}$, $h\mathord {\left( s\right) } = s^{-\frac{\theta }{1 - \theta }}$ and notice that *h* is monotonically decreasing as is the sequence $\mathord {\left( \sum _{k = n}^\infty \delta _k\right) }_{n \ge n_1}$. Therefore, for any $n\ge n_1$,
  $$\begin{aligned} 1&\le C_1^{\frac{\theta }{1 - \theta }} h\mathord {\left( \sum _{k = n+1}^\infty \delta _k\right) } \mathord {\left( \sum _{k = n}^\infty \delta _k - \sum _{k = n + 1}^\infty \delta _k\right) } \\&\le C_1^{\frac{\theta }{1 - \theta }} \int _{\sum _{k = n + 1}^\infty \delta _k}^{\sum _{k = n}^\infty \delta _k} h\mathord {\left( s\right) } \mathop {\mathrm {d} s} \\&= -C_1^{\frac{\theta }{1 - \theta }} \frac{1 - \theta }{2\theta - 1}\mathord {\left( \mathord {\left( \sum _{k = n}^\infty \delta _k\right) }^{-\frac{2\theta - 1}{1 - \theta }} - \mathord {\left( \sum _{k = n + 1}^\infty \delta _k\right) }^{-\frac{2\theta - 1}{1 - \theta }}\right) }. \end{aligned}$$
  Thus, by induction, for any $n\ge n_1+1$,
  $$\begin{aligned} \mathord {\left( \sum _{k = n}^\infty \delta _k\right) }^{-\frac{2\theta - 1}{1 - \theta }} + \frac{\mathord {\left( 2\theta - 1\right) }\mathord {\left( n - n_1\right) }}{C_1^{\frac{\theta }{1 - \theta }}\mathord {\left( 1 - \theta \right) }} \le \mathord {\left( \sum _{k = n_1}^\infty \delta _k\right) }^{-\frac{2\theta - 1}{1 - \theta }}. \end{aligned}$$
  The assertion follows by
  $$\begin{aligned} \delta _n\le & {} \sum _{k = n}^\infty \delta _k \le \mathord {\left( \mathord {\left( \sum _{k = n_1}^\infty \delta _k\right) }^{-\frac{2\theta - 1}{1 - \theta }} + \frac{\mathord {\left( 2\theta - 1\right) }\mathord {\left( n - n_1\right) }}{C_1\mathord {\left( 1 - \theta \right) }}\right) }^{-\frac{1 - \theta }{2\theta - 1}}\\&\text {for any } n \ge n_1+1. \end{aligned}$$
  $$\square $$

## Application to image processing

Consider an image of the size $m \times n$ pixels. (For the sake of simplicity, we consider gray-scale pictures only.) It can be represented by a vector $x \in \mathcal {H}\mathrel {\mathop :}=\mathbb {R}^{mn}$ of size *mn* with entries in $\left[ 0, 1\right]$ (where 0 represents pure black and 1 represents pure white).

The original image $x \in \mathcal {H}$ is assumed to be blurred by a linear operator $L: \mathcal {H}\rightarrow \mathcal {H}$ (e.g. the camera is out of focus or in movement during the exposure). Furthermore, it is corrupted with a noise $\nu$, so that only the result $b = Lx + \nu$ is known to us. We want to reconstruct the original image *x* by considering the minimisation problem

$$\begin{aligned} \min _{x \in \mathcal {H}} \mathord {\left( \frac{\mu }{2} \left\| Lx - b \right\| ^2 + J\mathord {\left( Dx\right) }\right) }, \end{aligned}$$

where we denote by $\left\| \cdot \right\|$ the usual Euclidean norm, $\mu > 0$ is a regularisation parameter, $D: \mathbb {R}^{mn} \rightarrow \mathbb {R}^{2mn}$ is the discrete gradient operator given by $Dx = \mathord {\left( K_1x, K_2x\right) }$, where

$$\begin{aligned} K_1: \mathcal {H}&\rightarrow \mathcal {H}, \mathord {\left( K_1 x\right) }_{i, j} \mathrel {\mathop :}={\left\{ \begin{array}{ll}x_{i + 1, j} - x_{i, j},&{} i = 1, \ldots , m-1; j = 1, \ldots , n; \\ 0,&{}\quad i = m; j = 1, \ldots , n\end{array}\right. } \\ K_2: \mathcal {H}&\rightarrow \mathcal {H}, \mathord {\left( K_2 x\right) }_{i, j} \mathrel {\mathop :}={\left\{ \begin{array}{ll}x_{i, j + 1} - x_{i, j},&{}\quad i = 1, \ldots , m; j = 1, \ldots , n - 1; \\ 0, &{}\quad i = 1,\ldots , m; j = n,\end{array}\right. } \end{aligned}$$

and $J: \mathcal {H}\rightarrow \mathbb {R}$ is a regularising functional penalising noisy images. We want to compare several choices of the functional *J* proposed by [1, 4], all of which have in common that they want to induce sparsity of *Dx*, i.e., having many components equal to zero.

The *Zhang penalty* [30] is defined by

$$\begin{aligned} \hbox {Zhang}_{\alpha }(z) = \sum \limits ^{2mn}_{j=1} g_\alpha (z_{j}), \end{aligned}$$

where $$\alpha > 0$$ and

$$\begin{aligned} g_{\alpha }({z_j})= & {} \left\{ \begin{array}{ll}\frac{1}{\alpha }|{z_j}| &{}\quad \text {if }\quad |{z_j}|< \alpha , \\ 1&{}\quad \text {if}\quad |z_{j}|\ge \alpha .\end{array}\right. \\= & {} \frac{1}{\alpha }{|{z_j}|} - \left\{ \begin{array}{ll}0 &{}\quad \text {if }\quad |{z_j}| < \alpha , \\ \frac{1}{\alpha }({|{z_j}|} - \alpha )&{}\quad \text {if }\quad |{z_j}| \ge \alpha . \end{array}\right. \end{aligned}$$

Denoting the part after the curly brace as $h_{\alpha }(z_{j})$ and $h_\alpha (z):=\sum \nolimits _{j=1}^{2mn} h_\alpha (z_j),$ we have

$$\begin{aligned} {{\mathrm{Prox}}}_{\gamma h_{\alpha }^*}\mathord {\left( z\right) } = \left\{ \begin{array}{ll}-\frac{1}{\alpha }&{}\quad \text {if }\quad z \le -\frac{1}{\alpha }-\gamma \alpha , \\ {z + \gamma {\alpha }} &{}\quad \text {if}\quad -\frac{1}{\alpha }-\gamma \alpha \le z \le -\gamma \alpha ,\\ 0&{}\quad \text {if}\quad -\gamma \alpha \le z \le \gamma \alpha ,\\ z-\gamma \alpha &{}\quad \text {if}\quad \gamma \alpha \le z \le \frac{1}{\alpha } +\gamma \alpha ,\\ \frac{1}{\alpha }&{}\quad \text {if}\quad z\ge \frac{1}{\alpha } +\gamma \alpha . \end{array}\right. \end{aligned}$$

The *LZOX penalty* [1] is defined by

$$\begin{aligned} {\mathrm {LZOX}}_{\alpha }\mathord {\left( z\right) } = \left\| Dx \right\| _{\ell ^1} - {\alpha } \left\| Dx \right\| _{\times }, \end{aligned}$$

where $\left\| \cdot \right\| _{\ell ^1}$ denotes (as usual) the sum of the absolute values and

$$\begin{aligned} \left\| \mathord {\left( u, v\right) } \right\| _\times \mathrel {\mathop :}=\sum _{i = 1}^m \sum _{j = 1}^n \sqrt{u_{i, j}^2 + v_{i, j}^2}, \end{aligned}$$

where $y = \mathord {\left( u, v\right) }$ is the splitting according to the definition of *D*. The algorithm (9)–(10) can now be applied to any of the models described above, since the models are written as d.c. problems and the components are easily accessible for computation, with the exception of the function $\left\| \cdot \right\| _{\ell ^1} \circ D$, see [31]. For the latter, see the following section.

**Table 1:** LZOX after 50 iterations

$\mu$	$\alpha = 0.00$	$\alpha = 0.2$	$\alpha = 0.4$	$\alpha = 0.5$	$\alpha = 0.6$	$\alpha = 0.8$	$\alpha = 1.0$
1.0	$-$ 3.0288	$-$ 4.2266	$-$ 3.7637	$-$ 3.6569	$-$3.5150	$-$4.3590	$-$13.701
10.0	5.9227	6.26615	6.414791	6.44871	6.45780	6.28863	4.301090
20.0	6.76613	6.90005	**6**.**93064**	6.917926	6.88018	6.61521	5.305623
50.0	6.81752	6.78308	6.65411	6.4923	6.36250	5.780558	4.741993
100.0	5.29597	5.23264	5.05189	4.91247	4.739717	4.287092	3.696120
200.0	3.088196	3.060511	2.985871	2.930448	2.863122	2.693096	2.477708
500.0	1.317390	1.312168	1.298834	1.288983	1.277010	1.246724	1.208036
1000.0	0.692487	0.691049	0.687585	0.685057	0.682000	0.674272	0.664401

**Table 2:** Zhang after 50 iterations

$\mu$	$\alpha = 0.01$	$\alpha = 0.03$	$\alpha = 0.1$	$\alpha = 0.3$	$\alpha = 1.0$	$\alpha = 3.0$
1.0	$-$ 43.708	$-$ 33.711	$-$ 23.148	$-$ 13.846	$-$3.0288	2.4922
10.0	$-$ 18.781	$-$ 9.9406	$-$ 3.2070	2.5442	5.9227	**6**.**97777**
20.0	$-$ 11.270	$-$ 4.8428	0.43533	4.7768	6.76613	6.57299
50.0	$-$ 4.8333	$-$ 1.05553	2.63959	6.46109	6.81752	3.952101
100.0	$-$ 1.7546	$-$ 0.14560	3.16532	6.90202	5.29597	2.129705
200.0	$-$ 0.41418	0.0619477	2.98543	6.38513	3.088196	1.110186
500.0	0.0077144	0.121807	2.101321	3.816813	1.317390	0.482406
1000.0	0.0528014	0.127592	1.423684	2.070959	0.692487	0.271777

**Fig. 2:**
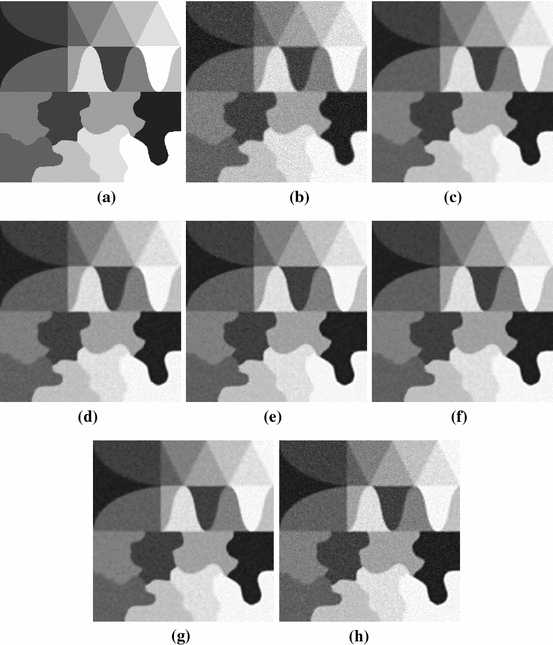
Original image, blurry and noisy image and reconstructions. **a** Original image. **b** Blurry image. **c** LZOX, $\mu = 20$, $\alpha = 0.4$. **d** LZOX, $\mu = 20$, $\alpha = 1$, **e** LZOX, $\mu = 50$, $\alpha = 0$, **f** Zhang, $\mu = 10$, $\alpha = 3$. **g** Zhang, $\mu = 20$, $\alpha = 1$. **h** Zhang, $\mu = 100$, $\alpha = 0.1$

**Fig. 3:**
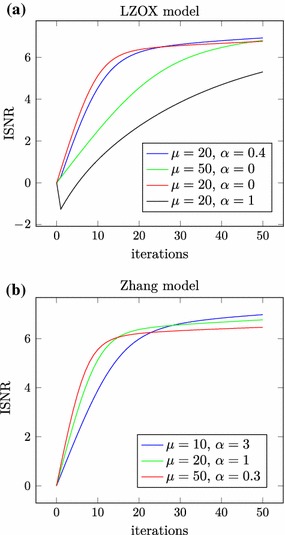
Improvement in signal-to-noise ratio versus iterations

### The proximal point of the anisotropic total variation

In order to apply Algorithm (9)–(10) to any of the problems, we have to calculate the proximal point of the anisotropic total variation by solving the optimisation problem

22 $$\begin{aligned} \inf \left\{ \frac{1}{2\gamma } \left\| x - b \right\| ^2 + \left\| Dx \right\| _{\ell ^1} \,\Big \vert \, x \in \mathcal {H} \right\} \end{aligned}$$

for some $\gamma > 0$ and $b \in \mathcal {H}$ in each step. The Fenchel dual problem [21, Chapter 19] is given by

23 $$\begin{aligned} \inf \left\{ \frac{\gamma }{2}\left\| D^* x^* \right\| ^2 - \left\langle b, D^* x^* \right\rangle \,\Big \vert \, x^* \in \mathcal {G}, \left\| x^* \right\| _{\ell ^\infty } \le 1 \right\} . \end{aligned}$$

Instead of solving (22), we could also solve (23) (see [32]), as the following result shows.

#### Lemma 5

Let $x^* \in \mathcal {G}$ be a solution of (23). Then $x = b - \gamma D^* x^*$ is a solution of (22).

#### Proof

See [21, Example 19.7]. In short:

$$\begin{aligned} 0 \in D\mathord {\left( \gamma D^* x^* - b\right) } + \partial \left\| \cdot \right\| _{\ell ^1}^*\mathord {\left( x^*\right) }&\implies D^* x^* \in D^* \partial \left\| \cdot \right\| _{\ell ^1}\mathord {\left( D\mathord {\left( b - \gamma D^* x^*\right) }\right) } \\&\implies \frac{1}{\gamma } \mathord {\left( b - x\right) } \in D^* \partial \left\| \cdot \right\| _{\ell ^1} \mathord {\left( Dx\right) }\\&\Longleftrightarrow 0 \in \partial \left( \frac{1}{2\gamma } \left\| (\cdot ) - b \right\| ^2 + \left\| D (\cdot ) \right\| _{\ell ^1} \right) (x). \end{aligned}$$

$$\square $$

To the formulation (23), the accelerated forward-backward algorithm of Beck and Teboulle [12] can be applied, since the objective function is differentiable and the feasible set is easy to project on.

### Numerical results

We implemented the FBDC algorithm applied to the model described above and tested the MATLAB code on a PC with Intel Core i5 4670S ($4\times$ 3.10 GHz) and 8 GB DDR3 RAM (1600 MHz). Our implementation used the method described in Sect. 5.1 until the $\ell ^\infty$ distance between two iterations was smaller than $10^{-4}$. Both stepsizes were chosen as $\mu _n = \gamma _n = \frac{1}{8\mu }$ for all $n\ge 0$. As initial value, we chose $x_0 = b$ and picked $v_0 \in \partial h\mathord {\left( Kx_0\right) }$.

We picked the image texmos3 from http://sipi.usc.edu/database/database.php?volume=textures&image=64 and convolved it with a Gaussian kernel with 9 pixels standard devitation. Afterwards we added white noise with standard deviation 50 / 255, projected the pixels back to the range $\left[ 0, 1\right]$ and saved the image in TIFF format, rounding the brightness values to multiples of 1 / 255. See Fig. 2 for original, blurry, and reconstructed image.

The *improvement in signal-to-noise ratio* or *ISNR value* of a reconstruction is given by

$$\begin{aligned} {\mathrm {ISNR}}\mathord {\left( x_k\right) } = 10 \log _{10} \mathord {\left( \frac{\left\| x - b \right\| ^2}{\left\| x - x_k \right\| ^2}\right) }, \end{aligned}$$

where *x* is the (usually unknown) original, *b* is the known blurry and noisy and $x_k$ is the reconstructed image. For the ISNR values after 50 iterations, see Tables 1 and 2. The development of the ISNR values over the iterations is shown in Fig. 3.

We see that the nonconvex models provide reasonable reconstructions of the original image and the best numerical performance for this particular choice of the stepsizes and the number of iterations is not achieved for the convex model (LZOX with $\alpha = 0$), but for the nonconvex models.
